# 3-Chloro-1-methyl-4-[2-(3-phenyl­allyl­idene)hydrazinyl­idene]-3,4-dihydro-1*H*-2λ^6^,1-benzothia­zine-2,2-dione

**DOI:** 10.1107/S1600536812051513

**Published:** 2013-01-04

**Authors:** Muhammad Shafiq, M. Nawaz Tahir, William T. A. Harrison, Islam Ullah Khan, Sidra Shafique

**Affiliations:** a Department of Chemistry, Government College University, Faisalabad 38000, Pakistan; b Department of Physics, University of Sargodha, Sargodha, Pakistan; c Department of Chemistry, University of Aberdeen, Mston Walk, Aberdeen AB24 3UE, Scotland; d Materials Chemistry Laboratory, Department of Chemistry, Government College University, Lahore, Pakistan

## Abstract

In the title compound, C_18_H_16_ClN_3_O_2_S, the dihedral angle between the aromatic rings is 4.81 (2)° and the alkyl chain takes on an extended conformation [N—C—C—C = 179.2 (4)°]. The conformation of the thia­zine ring is an envelope, with the S atom displaced by −0.805 (3) Å from the mean plane of the other five atoms (r.m.s. deviation = 0.046 Å). The Cl atom is an axial conformation and is displaced by 1.761 (4) Å from the thia­zine ring plane. In the crystal, inversion dimers linked by pairs of C—H⋯O inter­actions generate *R*_2_^2^(20) loops and further C—H⋯O hydrogen bonds link the dimers into (001) sheets. Weak aromatic π–π stacking inter­actions [centroid–centroid separations = 3.870 (3) and 3.883 (3) Å] are also observed.

## Related literature

For the synthesis and biological activity of the title compound and related materials, see: Shafiq *et al.* (2011*a* ▶). For further synthetic details, see: Shafiq *et al.* (2011*b* ▶). 
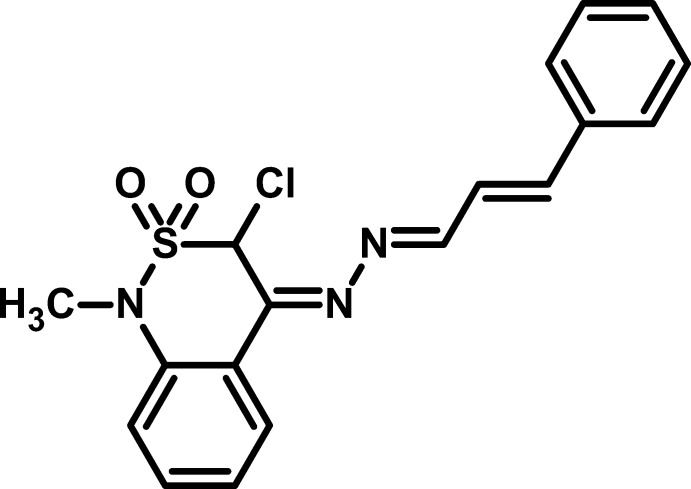


## Experimental

### 

#### Crystal data

C_18_H_16_ClN_3_O_2_S
*M* *_r_* = 373.85
Monoclinic, 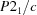

*a* = 7.2262 (5) Å
*b* = 13.5823 (9) Å
*c* = 17.9818 (12) Å
β = 97.023 (4)°
*V* = 1751.6 (2) Å^3^
*Z* = 4
Mo *K*α radiation
μ = 0.35 mm^−1^
*T* = 296 K
0.32 × 0.20 × 0.18 mm

#### Data collection

Bruker APEXII CCD diffractometer
Absorption correction: multi-scan (*SADABS*; Bruker, 2007 ▶) *T*_min_ = 0.895, *T*_max_ = 0.939
8051 measured reflections
3107 independent reflections
1808 reflections with *I* > 2σ(*I*)
*R*_int_ = 0.043

#### Refinement

*R*[*F* ^2^ > 2σ(*F* ^2^)] = 0.067
*wR*(*F* ^2^) = 0.201
*S* = 1.03
3107 reflections
227 parameters
H-atom parameters constrained
Δρ_max_ = 0.97 e Å^−3^
Δρ_min_ = −0.36 e Å^−3^

### 

Data collection: *APEX2* (Bruker, 2007 ▶); cell refinement: *SAINT* (Bruker, 2007 ▶); data reduction: *SAINT*; program(s) used to solve structure: *SHELXS97* (Sheldrick, 2008 ▶); program(s) used to refine structure: *SHELXL97* (Sheldrick, 2008 ▶); molecular graphics: *ORTEP-3* (Farrugia, 2012 ▶); software used to prepare material for publication: *SHELXL97*.

## Supplementary Material

Crystal structure: contains datablock(s) global, I. DOI: 10.1107/S1600536812051513/bq2381sup1.cif

Structure factors: contains datablock(s) I. DOI: 10.1107/S1600536812051513/bq2381Isup2.hkl

Supplementary material file. DOI: 10.1107/S1600536812051513/bq2381Isup3.cml

Additional supplementary materials:  crystallographic information; 3D view; checkCIF report

## Figures and Tables

**Table 1:** Hydrogen-bond geometry (Å, °)

*D*—H⋯*A*	*D*—H	H⋯*A*	*D*⋯*A*	*D*—H⋯*A*
C12—H12⋯O2^i^	0.93	2.51	3.345 (6)	150
C15—H15⋯O2^ii^	0.93	2.57	3.464 (6)	161

Symmetry codes: (i) 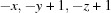; (ii) 
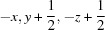
.

**Fig. 1.**
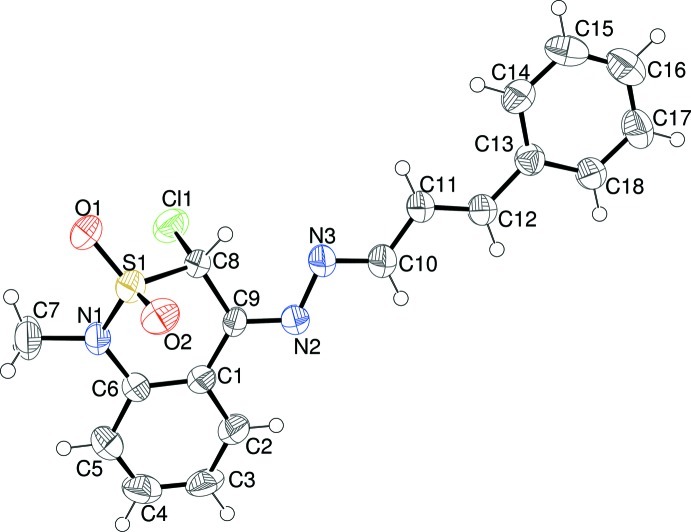
The molecular structure of (I), showing displacement ellipsoids at the 50% probability level.

## supplementary crystallographic information

### Comment

As part of our ongoing studies of benzothiazine derivatives with potential biactivity (Shafiq *et al.*, 2011*a*,*b*), we now describe the synthesis and structure of the title compound, (I).

The dihedral angle between the C1–C6 and C13–C18 aromatic rings is 4.81 (2)° and the C9=N2—N3=C10 torsion angle is -178.1 (4)°. The linking alkyl chain takes on an extended conformation [N3—C10—C11—C12 = 179.2 (4)°]. The conformation of the C1/C6/C8/C9/N1/S1 thiazine ring is an envelope, with the S atom displaced by -0.805 (3) Å from the mean plane of the other five atoms (r.m.s. deviation = 0.046 Å). Atom C16 is displaced from the mean plane by 0.343 (6) Å and Cl1, in an axial site, is displaced by 1.761 (4) Å. Atom C8 is a stereogenic centre (*R* configuration in the arbitrarily-chosen asymmetric unit), but crystal symmetry generates a racemic mixture.

In the crystal, inversion dimers linked by pairs of C—H···O interactions (Table 1) to generate *R*_2_^2^(20) loops. Further C—H···O bonds link the dimers into (001) sheets. Weak aromatic π-π stacking interactions between the C1—C6 and C13—C18 benzene rings [centroid-centroid separations = 3.870 (3) and 3.883 (3) Å] are also observed.

### Experimental

The Schiff base derivative of (4*Z*)-4-hydrazinylidene-1-methyl-3,4-dihydro -1*H*-2,1-benzothiazine 2,2-dioxide and *trans* cinnamaldehyde was prepared using the methods reported previously (Shafiq *et al.*, 2011*a*). The chlorination of the Schiff base was undertaken using *N*-chloro succinimide and dibenzoylperoxide (Shafiq *et al.*, 2011*b*). The crude product was recrystallized from ethyl acetate solution to obtain yellow blocks of the title compound.

### Refinement

The H atoms were placed in calculated positions (C—H = 0.93–0.97 Å) and refined as riding. The methyl group was allowed to rotate, but not to tip, to best fit the electron density. The constraint *U*_iso_(H) = 1.2*U*_eq_(C) or 1.5*U*_eq_(methyl C) was applied.

### Crystal data

**Table:**

C_18_H_16_ClN_3_O_2_S	*F*(000) = 776
*M**_r_* = 373.85	*D*_x_ = 1.418 Mg m^−^^3^
Monoclinic, *P*2_1_/*c*	Mo *K*α radiation, λ = 0.71073 Å
Hall symbol: -P 2ybc	Cell parameters from 305 reflections
*a* = 7.2262 (5) Å	θ = 3.5–24.5°
*b* = 13.5823 (9) Å	µ = 0.35 mm^−^^1^
*c* = 17.9818 (12) Å	*T* = 296 K
β = 97.023 (4)°	Rod, yellow
*V* = 1751.6 (2) Å^3^	0.32 × 0.20 × 0.18 mm
*Z* = 4	

### Data collection

**Table:**

Bruker APEXII CCD diffractometer	3107 independent reflections
Radiation source: fine-focus sealed tube	1808 reflections with *I* > 2σ(*I*)
Graphite monochromator	*R*_int_ = 0.043
ω scans	θ_max_ = 25.5°, θ_min_ = 2.7°
Absorption correction: multi-scan (*SADABS*; Bruker, 2007)	*h* = −8→8
*T*_min_ = 0.895, *T*_max_ = 0.939	*k* = −13→16
8051 measured reflections	*l* = −21→18

### Refinement

**Table:**

Refinement on *F*^2^	Primary atom site location: structure-invariant direct methods
Least-squares matrix: full	Secondary atom site location: difference Fourier map
*R*[*F*^2^ > 2σ(*F*^2^)] = 0.067	Hydrogen site location: inferred from neighbouring sites
*wR*(*F*^2^) = 0.201	H-atom parameters constrained
*S* = 1.03	*w* = 1/[σ^2^(*F*_o_^2^) + (0.1036*P*)^2^ + 0.2998*P*] where *P* = (*F*_o_^2^ + 2*F*_c_^2^)/3
3107 reflections	(Δ/σ)_max_ < 0.001
227 parameters	Δρ_max_ = 0.97 e Å^−^^3^
0 restraints	Δρ_min_ = −0.36 e Å^−^^3^

### Special details

**Table:**

Geometry. All e.s.d.'s (except the e.s.d. in the dihedral angle between two l.s. planes) are estimated using the full covariance matrix. The cell e.s.d.'s are taken into account individually in the estimation of e.s.d.'s in distances, angles and torsion angles; correlations between e.s.d.'s in cell parameters are only used when they are defined by crystal symmetry. An approximate (isotropic) treatment of cell e.s.d.'s is used for estimating e.s.d.'s involving l.s. planes.
Refinement. Refinement of *F*^2^ against ALL reflections. The weighted *R*-factor *wR* and goodness of fit *S* are based on *F*^2^, conventional *R*-factors *R* are based on *F*, with *F* set to zero for negative *F*^2^. The threshold expression of *F*^2^ > σ(*F*^2^) is used only for calculating *R*-factors(gt) *etc*. and is not relevant to the choice of reflections for refinement. *R*-factors based on *F*^2^ are statistically about twice as large as those based on *F*, and *R*- factors based on ALL data will be even larger.

### Fractional atomic coordinates and isotropic or equivalent isotropic displacement parameters (Å^2^)

**Table:**

	*x*	*y*	*z*	*U*_iso_*/*U*_eq_	
C1	0.2953 (5)	0.2779 (3)	0.6118 (2)	0.0406 (11)	
C2	0.3170 (6)	0.3399 (4)	0.6745 (3)	0.0526 (12)	
H2	0.3146	0.4077	0.6673	0.063*	
C3	0.3415 (7)	0.3037 (4)	0.7460 (3)	0.0601 (14)	
H3	0.3549	0.3465	0.7867	0.072*	
C4	0.3463 (7)	0.2037 (4)	0.7574 (3)	0.0622 (14)	
H4	0.3660	0.1791	0.8059	0.075*	
C5	0.3225 (7)	0.1399 (4)	0.6983 (3)	0.0584 (13)	
H5	0.3231	0.0724	0.7069	0.070*	
C6	0.2973 (6)	0.1759 (3)	0.6248 (2)	0.0422 (11)	
C7	0.3297 (8)	0.0041 (3)	0.5761 (3)	0.0762 (17)	
H7A	0.4520	0.0003	0.6036	0.114*	
H7B	0.3302	−0.0271	0.5282	0.114*	
H7C	0.2419	−0.0286	0.6036	0.114*	
C8	0.2780 (6)	0.2531 (3)	0.4704 (3)	0.0422 (11)	
H8	0.2142	0.2856	0.4257	0.051*	
C9	0.2729 (5)	0.3201 (3)	0.5368 (2)	0.0381 (10)	
C10	0.2243 (6)	0.5395 (3)	0.4480 (3)	0.0507 (12)	
H10	0.2233	0.5762	0.4916	0.061*	
C11	0.2092 (6)	0.5897 (3)	0.3787 (3)	0.0489 (12)	
H11	0.2116	0.5542	0.3345	0.059*	
C12	0.1917 (6)	0.6871 (3)	0.3759 (3)	0.0480 (12)	
H12	0.1893	0.7181	0.4219	0.058*	
C13	0.1756 (6)	0.7522 (3)	0.3107 (3)	0.0450 (11)	
C14	0.1646 (7)	0.7194 (4)	0.2366 (3)	0.0563 (13)	
H14	0.1665	0.6522	0.2266	0.068*	
C15	0.1508 (7)	0.7866 (5)	0.1777 (3)	0.0634 (14)	
H15	0.1434	0.7639	0.1287	0.076*	
C16	0.1482 (7)	0.8848 (5)	0.1912 (3)	0.0683 (15)	
H16	0.1402	0.9291	0.1515	0.082*	
C17	0.1573 (7)	0.9191 (4)	0.2634 (3)	0.0638 (14)	
H17	0.1536	0.9864	0.2725	0.077*	
C18	0.1717 (6)	0.8543 (3)	0.3220 (3)	0.0517 (12)	
H18	0.1791	0.8786	0.3706	0.062*	
S1	0.15957 (18)	0.14181 (8)	0.48575 (7)	0.0514 (4)	
O1	0.1890 (5)	0.0698 (2)	0.42999 (19)	0.0666 (10)	
O2	−0.0251 (4)	0.1739 (2)	0.49385 (18)	0.0542 (9)	
N1	0.2763 (5)	0.1075 (3)	0.5651 (2)	0.0518 (10)	
N2	0.2523 (5)	0.4134 (3)	0.5278 (2)	0.0478 (10)	
N3	0.2393 (5)	0.4455 (3)	0.4536 (2)	0.0526 (10)	
Cl1	0.51237 (17)	0.22812 (9)	0.45547 (8)	0.0621 (4)	

### Atomic displacement parameters (Å^2^)

**Table:**

	*U*^11^	*U*^22^	*U*^33^	*U*^12^	*U*^13^	*U*^23^
C1	0.043 (2)	0.041 (3)	0.039 (3)	0.0041 (18)	0.0078 (19)	−0.002 (2)
C2	0.064 (3)	0.050 (3)	0.044 (3)	0.006 (2)	0.007 (2)	−0.006 (3)
C3	0.071 (3)	0.075 (4)	0.034 (3)	0.009 (3)	0.006 (2)	−0.016 (3)
C4	0.066 (3)	0.082 (4)	0.038 (3)	0.007 (3)	0.007 (2)	0.009 (3)
C5	0.071 (3)	0.054 (3)	0.049 (3)	0.006 (2)	0.004 (2)	0.013 (3)
C6	0.049 (3)	0.040 (3)	0.038 (3)	0.0043 (19)	0.006 (2)	0.004 (2)
C7	0.116 (5)	0.035 (3)	0.074 (4)	0.008 (3)	−0.004 (3)	0.004 (3)
C8	0.058 (3)	0.032 (2)	0.037 (3)	0.0042 (18)	0.009 (2)	−0.001 (2)
C9	0.045 (2)	0.028 (2)	0.041 (3)	0.0045 (17)	0.0049 (19)	−0.003 (2)
C10	0.063 (3)	0.038 (3)	0.051 (3)	0.002 (2)	0.006 (2)	0.000 (2)
C11	0.065 (3)	0.036 (3)	0.047 (3)	0.001 (2)	0.010 (2)	0.002 (2)
C12	0.060 (3)	0.038 (3)	0.046 (3)	−0.003 (2)	0.010 (2)	0.000 (2)
C13	0.046 (3)	0.042 (3)	0.046 (3)	−0.0007 (18)	0.003 (2)	0.000 (2)
C14	0.065 (3)	0.049 (3)	0.055 (4)	−0.001 (2)	0.008 (2)	−0.009 (3)
C15	0.061 (3)	0.088 (4)	0.039 (3)	−0.009 (3)	0.001 (2)	0.002 (3)
C16	0.065 (3)	0.080 (4)	0.061 (4)	−0.002 (3)	0.010 (3)	0.024 (3)
C17	0.072 (3)	0.046 (3)	0.073 (4)	−0.003 (2)	0.011 (3)	0.016 (3)
C18	0.065 (3)	0.042 (3)	0.050 (3)	−0.003 (2)	0.013 (2)	0.003 (2)
S1	0.0683 (9)	0.0383 (7)	0.0475 (8)	−0.0027 (5)	0.0070 (6)	−0.0040 (6)
O1	0.109 (3)	0.0401 (19)	0.052 (2)	−0.0042 (17)	0.0127 (19)	−0.0149 (17)
O2	0.0472 (19)	0.060 (2)	0.055 (2)	−0.0051 (14)	0.0033 (15)	−0.0113 (17)
N1	0.081 (3)	0.031 (2)	0.042 (3)	0.0058 (17)	0.0017 (19)	0.0016 (19)
N2	0.066 (3)	0.038 (2)	0.039 (2)	0.0037 (16)	0.0049 (18)	0.0004 (18)
N3	0.076 (3)	0.035 (2)	0.046 (3)	0.0026 (18)	0.0063 (19)	0.0018 (19)
Cl1	0.0630 (8)	0.0605 (8)	0.0664 (10)	0.0036 (5)	0.0223 (6)	−0.0108 (7)

### Geometric parameters (Å, º)

**Table:**

C1—C2	1.401 (6)	C10—C11	1.414 (6)
C1—C6	1.405 (6)	C10—H10	0.9300
C1—C9	1.455 (6)	C11—C12	1.329 (6)
C2—C3	1.368 (7)	C11—H11	0.9300
C2—H2	0.9300	C12—C13	1.462 (6)
C3—C4	1.373 (7)	C12—H12	0.9300
C3—H3	0.9300	C13—C14	1.398 (7)
C4—C5	1.365 (7)	C13—C18	1.402 (6)
C4—H4	0.9300	C14—C15	1.393 (7)
C5—C6	1.399 (6)	C14—H14	0.9300
C5—H5	0.9300	C15—C16	1.357 (7)
C6—N1	1.415 (6)	C15—H15	0.9300
C7—N1	1.463 (6)	C16—C17	1.373 (7)
C7—H7A	0.9600	C16—H16	0.9300
C7—H7B	0.9600	C17—C18	1.366 (6)
C7—H7C	0.9600	C17—H17	0.9300
C8—C9	1.506 (6)	C18—H18	0.9300
C8—S1	1.775 (4)	S1—O2	1.428 (3)
C8—Cl1	1.780 (4)	S1—O1	1.435 (3)
C8—H8	0.9800	S1—N1	1.634 (4)
C9—N2	1.283 (5)	N2—N3	1.397 (5)
C10—N3	1.284 (5)		
			
C2—C1—C6	117.4 (4)	C12—C11—C10	120.7 (5)
C2—C1—C9	119.8 (4)	C12—C11—H11	119.7
C6—C1—C9	122.8 (4)	C10—C11—H11	119.7
C3—C2—C1	122.0 (5)	C11—C12—C13	129.1 (5)
C3—C2—H2	119.0	C11—C12—H12	115.4
C1—C2—H2	119.0	C13—C12—H12	115.4
C2—C3—C4	119.5 (5)	C14—C13—C18	117.0 (4)
C2—C3—H3	120.2	C14—C13—C12	124.0 (4)
C4—C3—H3	120.2	C18—C13—C12	119.0 (4)
C5—C4—C3	120.9 (5)	C15—C14—C13	120.4 (5)
C5—C4—H4	119.5	C15—C14—H14	119.8
C3—C4—H4	119.5	C13—C14—H14	119.8
C4—C5—C6	120.2 (5)	C16—C15—C14	120.7 (5)
C4—C5—H5	119.9	C16—C15—H15	119.7
C6—C5—H5	119.9	C14—C15—H15	119.7
C5—C6—C1	120.0 (4)	C15—C16—C17	120.2 (5)
C5—C6—N1	118.5 (4)	C15—C16—H16	119.9
C1—C6—N1	121.5 (4)	C17—C16—H16	119.9
N1—C7—H7A	109.5	C18—C17—C16	120.0 (5)
N1—C7—H7B	109.5	C18—C17—H17	120.0
H7A—C7—H7B	109.5	C16—C17—H17	120.0
N1—C7—H7C	109.5	C17—C18—C13	121.8 (5)
H7A—C7—H7C	109.5	C17—C18—H18	119.1
H7B—C7—H7C	109.5	C13—C18—H18	119.1
C9—C8—S1	109.4 (3)	O2—S1—O1	120.0 (2)
C9—C8—Cl1	110.5 (3)	O2—S1—N1	112.8 (2)
S1—C8—Cl1	110.3 (2)	O1—S1—N1	108.1 (2)
C9—C8—H8	108.9	O2—S1—C8	103.3 (2)
S1—C8—H8	108.9	O1—S1—C8	110.9 (2)
Cl1—C8—H8	108.9	N1—S1—C8	99.9 (2)
N2—C9—C1	120.3 (4)	C6—N1—C7	121.7 (4)
N2—C9—C8	120.7 (4)	C6—N1—S1	118.1 (3)
C1—C9—C8	119.1 (4)	C7—N1—S1	119.4 (3)
N3—C10—C11	123.1 (5)	C9—N2—N3	115.0 (4)
N3—C10—H10	118.5	C10—N3—N2	112.4 (4)
C11—C10—H10	118.5		
			
C6—C1—C2—C3	0.8 (6)	C14—C15—C16—C17	−0.7 (8)
C9—C1—C2—C3	−178.9 (4)	C15—C16—C17—C18	0.9 (8)
C1—C2—C3—C4	0.4 (7)	C16—C17—C18—C13	−0.7 (7)
C2—C3—C4—C5	−1.5 (7)	C14—C13—C18—C17	0.2 (7)
C3—C4—C5—C6	1.5 (7)	C12—C13—C18—C17	179.9 (4)
C4—C5—C6—C1	−0.4 (7)	C9—C8—S1—O2	60.3 (3)
C4—C5—C6—N1	178.9 (4)	Cl1—C8—S1—O2	−178.0 (2)
C2—C1—C6—C5	−0.8 (6)	C9—C8—S1—O1	−170.0 (3)
C9—C1—C6—C5	178.9 (4)	Cl1—C8—S1—O1	−48.2 (3)
C2—C1—C6—N1	180.0 (4)	C9—C8—S1—N1	−56.2 (3)
C9—C1—C6—N1	−0.4 (6)	Cl1—C8—S1—N1	65.6 (3)
C2—C1—C9—N2	−7.8 (6)	C5—C6—N1—C7	−16.9 (6)
C6—C1—C9—N2	172.6 (4)	C1—C6—N1—C7	162.4 (4)
C2—C1—C9—C8	171.1 (4)	C5—C6—N1—S1	153.4 (3)
C6—C1—C9—C8	−8.5 (6)	C1—C6—N1—S1	−27.4 (5)
S1—C8—C9—N2	−142.1 (3)	O2—S1—N1—C6	−56.8 (4)
Cl1—C8—C9—N2	96.3 (4)	O1—S1—N1—C6	168.2 (3)
S1—C8—C9—C1	39.1 (5)	C8—S1—N1—C6	52.3 (4)
Cl1—C8—C9—C1	−82.5 (4)	O2—S1—N1—C7	113.7 (4)
N3—C10—C11—C12	179.2 (4)	O1—S1—N1—C7	−21.3 (4)
C10—C11—C12—C13	179.5 (4)	C8—S1—N1—C7	−137.2 (4)
C11—C12—C13—C14	4.2 (7)	C1—C9—N2—N3	177.7 (3)
C11—C12—C13—C18	−175.5 (5)	C8—C9—N2—N3	−1.2 (5)
C18—C13—C14—C15	0.1 (7)	C11—C10—N3—N2	180.0 (4)
C12—C13—C14—C15	−179.5 (4)	C9—N2—N3—C10	−178.1 (4)
C13—C14—C15—C16	0.1 (7)		

### Hydrogen-bond geometry (Å, º)

**Table:**

*D*—H···*A*	*D*—H	H···*A*	*D*···*A*	*D*—H···*A*
C12—H12···O2^i^	0.93	2.51	3.345 (6)	150
C15—H15···O2^ii^	0.93	2.57	3.464 (6)	161

Symmetry codes: (i) −*x*, −*y*+1, −*z*+1; (ii) −*x*, *y*+1/2, −*z*+1/2.
